# Work-related musculoskeletal disorders in the automotive industry: a state-of-the-art literature review

**DOI:** 10.47626/1679-4435-2026-1462

**Published:** 2026-02-12

**Authors:** Igor da Silva Pereira, Igor Cunha Trindade, Karen Blenda Bonfim Correia, Vanessa Aparecida de Almeida, Eliana Napoleão Cozendey-Silva

**Affiliations:** 1 Universidade Federal de Catalão, Instituto de Biotecnologia, Departamento de Medicina, Catalão, GO, Brasil; 2 Fundação Oswaldo Cruz, Escola Nacional de Saúde Pública Sergio Arouca, Centro de Estudos da Saúde do Trabalhador e Ecologia Humana, Rio de Janeiro, RJ, Brasil

**Keywords:** work-related musculoskeletal disorders, industry, automobiles, workers’, health, occupational health surveillance., distúrbio osteomuscular relacionado ao trabalho, indústria, automóveis, saúde do trabalhador, vigilância em saúde do trabalhador.

## Abstract

Work-related musculoskeletal disorders are occupational conditions that result from the excessive use of the musculoskeletal system, combined with insufficient time for physiological recovery. These disorders remain prevalent in the automotive industry, particularly on assembly lines, and directly impact workers’ health and sector productivity. This study aimed to analyze recent scientific literature on work-related musculoskeletal disorders in the automotive industry. The analysis focused on research approaches, preventive practices, identified challenges, and future directions. The study covered the period from 2020 to 2025 and used a state-of-the-art literature review method. The literature was considered an interpretive and contextual field. Searches were conducted in the SciELO, LILACS, PubMed, and BVS databases using free-text and controlled terms combined with Boolean operators and adapted to the specificities of each database. Grey literature was included through Google Scholar. Study selection was conducted in successive stages: screening, full-text reading, and critical analysis guided by research questions. These stages followed predefined eligibility criteria. Of the 297 initial records, six studies met the established criteria. Repetitive movements, static postures, and production pace remain central determinants of the incidence and prevalence of work-related musculoskeletal disorders. The findings underscore the importance of integrating technological development and innovation with the active involvement of workers to build safer, healthier, and more sustainable work environments. Promoting network-based research in which workers play a central role is key to advancing solutions that align with occupational health surveillance guidelines and contemporary workplace challenges.

## Abstract (pt)

Os distúrbios osteomusculares relacionados ao trabalho configuram agravos ocupacionais decorrentes do uso excessivo do sistema musculoesquelético, associado à ausência de tempo adequado para recuperação fisiológica. Esses agravos permanecem frequentes na indústria automotiva, especialmente nas linhas de montagem, com impactos diretos sobre a saúde dos trabalhadores e a produtividade do setor. Este estudo teve como objetivo analisar a produção científica recente acerca dos distúrbios osteomusculares relacionados ao trabalho na indústria automotiva, com ênfase nas abordagens adotadas, nas práticas preventivas, nos desafios identificados e nas direções futuras, no período de 2020 a 2025, por meio de uma revisão da literatura do tipo estado da arte. A literatura foi considerada como campo interpretativo e contextual. A busca foi realizada nas bases SciELO, LILACS, PubMed e BVS utilizando termos livres e controlados, combinados por operadores booleanos e adaptados às especificidades de cada base. Incluiu-se literatura cinzenta por meio do Google Scholar. A seleção dos estudos seguiu critérios de elegibilidade e ocorreu em etapas sucessivas: triagem, leitura na íntegra e análise crítica orientada por questões de pesquisa. Dos 297 registros inicialmente identificados, seis estudos atenderam aos critérios estabelecidos. Movimentos repetitivos, posturas estáticas e o ritmo produtivo mantêm-se como determinantes centrais da incidência e prevalência dos distúrbios osteomusculares relacionados ao trabalho. Evidencia-se a necessidade de pesquisas que integrem o desenvolvimento e a inovação tecnológica à participação ativa dos trabalhadores, como estratégia para a construção de ambientes de trabalho mais seguros, saudáveis e sustentáveis. A promoção de pesquisas em rede, com protagonismo dos trabalhadores, mostra-se estratégica para o avanço de soluções alinhadas às diretrizes em vigilância em saúde do trabalhador e aos desafios contemporâneos do mundo do trabalho.

## INTRODUCTION

Repetitive strain injuries (RSIs) and work-related musculoskeletal disorders (WMSDs) are occupational conditions resulting from excessive use of the musculoskeletal system combined with insufficient time for physiological recovery. These conditions favor the development of symptoms such as pain, a sensation of heaviness, fatigue, functional limitation, and paresthesia. They are often accompanied by psychological distress, difficulties in performing activities of daily living, and work-related disability [^1^]. In Brazil, RSIs and WMSDs are subject to compulsory notification [^1^] and represent a major occupational health problem, with 6,582 affected workers reported between 2019 and 2024 [^2^]. Beyond their direct impact on the working population, these conditions lead to productivity losses and substantial economic implications for employers, governments, and society, underscoring the need for preventive strategies and qualified care [^3^].

According to the European Agency for Safety and Health at Work [^4^], WMSDs remain the most common work-related health problem and one of the greatest occupational challenges faced by companies. The Sixth European Working Conditions Survey (2015), published in 2017 [^5^], shows that postural risks, particularly repetitive movements of the hands and arms, are the most prevalent and affect approximately 61% of workers. In addition, the 2020 report indicates that 43% of workers reported back pain and 41% reported muscular pain in the shoulders, neck, and/or upper or lower limbs. These figures vary widely across European Union Member States, ranging from 79% in Finland to 40% in Hungary for self-reported low back pain [^4^].

The occurrence of WMSDs is not limited to the European context. In the automotive industry, the organization of work itself favors their incidence [^5^]. Tasks performed on production lines routinely involve repetitive movements, such as tightening, grasping, and handling materials. The continuous execution of these activities overloads the musculature and increases the risk of musculoskeletal illness [^6^]. In 2023, the automotive industry accounted for approximately 20% of Brazil’s gross domestic product [^7^], highlighting its substantial economic relevance and, simultaneously, its implications for workers’ health. This scenario reinforces the need for ongoing Worker Health Surveillance (Visat for short, in Portuguese) actions and preventive measures.

Worker Health is a field within Collective Health that integrates interdisciplinary practices and knowledge of a social, technical, political, and human nature, involving multiprofessional and interinstitutional action [^8^]. Its focus is the promotion, protection, and rehabilitation of workers’ health. As established by the National Worker and Worker’s Health Policy (PNSTT for short, in Portuguese) [^9^], Visat actions should be developed continuously and systematically, with the aim of detecting, understanding, investigating, and analyzing the determinants and conditioning factors of health problems related to work processes and environments. In the automotive sector, these strategies play a central role in addressing WMSDs, given the specific characteristics of work organization and the ergonomic risks involved.

This study emerged from reflections developed within the context of Health Care in Community Practice - Worker Health, linked to the medical course of a federal university in Southeast Goiás. An analysis of working conditions on assembly lines in the automotive industry of the Center-Southeast macroregion of Goiás revealed that WMSDs remain recurrent conditions, with significant repercussions for both workers’ health and regional economic dynamics.

Because of the persistence of WMSDs in the automotive sector and their ongoing impact on workers’ health, this study aims to analyze scientific production from the past 5 years on this topic, with emphasis on understanding WMSDs in critical areas such as assembly lines. The study seeks to address the following questions: how has recent scientific literature approached the challenges associated with WMSDs in the automotive industry? Which prevention and management practices have proven most effective, and what future directions have been highlighted by research in this field?

## METHODS

This study is a state-of-the-art (SotA) literature review, as outlined by Barry et al. [^10^]. A SotA review is grounded in the premise that there is no single, objectively true synthesis of a body of literature. It assumes that scientific production is open to interpretation and that the context in which the review is conducted shapes the resulting synthesis. Accordingly, the literature is understood as an interpretative and contextual field, capable of revealing different pathways and trends within the investigated domain.

The adopted methodology [^11^] comprised six interdependent stages: i) definition of the scope and formulation of the guiding review questions; ii) systematic search and careful selection of relevant literature, with emphasis on diversity and depth of studies; iii) reading of the texts and interpretative data extraction; iv) critical analysis and construction of meaning based on the identified evidence; v) organization of the narrative synthesis, highlighting advances, tensions, and gaps in the field; and vi) manuscript preparation, with attention to clarity, internal coherence, and theoretical contribution. These stages were applied iteratively and reflexively, allowing for a progressive deepening of understanding throughout the review process.

Regarding search systematization, the review was guided by two review questions and conducted in the following databases and search platforms: SciELO, PubMed, LILACS, and BVS, covering the period from January 2020 to January 2025. Grey literature was identified through Google Scholar.

The selection of free-text and controlled terms was guided by the review questions and preceded by a preliminary exploration of the literature. The terms were subsequently combined using Boolean operators (AND, OR, and NOT), including synonyms and English-language equivalents, with adaptations to the specific characteristics of each database or search platform (Table 1) to enhance search sensitivity. Studies addressing other industrial sectors, those that did not contribute to answering the research questions, or those lacking a clearly described methodology were excluded.

**Table 1:** Search strategies and number of articles retrieved by database (2020-2025)

Databases/search platforms	Search strategy	Articles retrieved
SciELO/LILACS/BVS	#01 (“Cumulative Trauma Disorders” OR “Distúrbios Osteomusculares” OR “LER” OR “Musculoskeletal Disorders”) AND (“Automóveis” OR “Indústria Automobilística” OR “Montadoras” OR “Automobiles” OR “Automotive Industry”) AND (“Saúde Ocupacional” OR “Occupational Health”) #02 (“Vigilância em Saúde do Trabalhador” OR “Surveillance of the Workers Health”) #03 ((year_cluster:[2020 TO 2025])	10/38/54
PubMed	((“musculoskeletal disorders”[TIAB] OR “work-related musculoskeletal disorders”[TIAB] OR “WMSDs”[TIAB] OR “cumulative trauma disorders”[TIAB]) AND #02 (“automotive industry”[TIAB] OR “automobile manufacturing”[TIAB] OR “automobile industry”[TIAB] OR “automotive manufacturing”[TIAB) AND #03 (“occupational health”[TIAB] OR “occupational exposure”[TIAB] OR “health risk assessment”[TIAB] OR “occupational risk assessment”[TIAB] OR “control strategies”[TIAB] OR “key control measures”[TIAB]) AND #04 (prevalence[TIAB] OR “influencing factors”[TIAB] OR “systematic review”[Publication Type] OR “meta-analysis”[Publication Type])) AND (“2020/01/01”[Date - Publication] : “2025/01/01”[Date - Publication])	8
Google Scholar	“riscos ergonômicos” AND (“linha de produção” OR “indústria automotiva”)	222

Eligibility criteria included studies published in any language that addressed WMSDs among workers in the automotive industry, considering variables such as prevalence, risk factors, and health impacts. Studies focusing on other industrial sectors were excluded, as were those lacking thematic alignment with the object of study, such as individual clinical case reports and conference abstracts.

Study selection was conducted in sequential stages, beginning with title and abstract screening, followed by full-text reading of potentially eligible articles and application of the eligibility criteria. All records retrieved during the search process were organized and managed using Zotero^®^ software, version 5.0.

Data extraction and analysis were performed independently by two researchers (IP and ECS). Discrepancies were resolved by consensus, resulting in an interrater agreement exceeding 90%. After validation of the interpretations, the analysis focused on identifying textual elements that addressed the research questions. These elements included findings related to incidence and prevalence, factors associated with WMSDs in the automotive sector, as well as adopted practices, identified challenges, and future directions highlighted in the literature.

## RESULTS

Of the 332 articles initially identified (Table 1), 35 were excluded due to duplication. Among the remaining 297 studies, 6 met the eligibility criteria.

Table 2 presents a synthesis of the main findings of the literature on WMSDs in the automotive industry over the past 5 years.

**Table 2:** Synthesis and main findings of studies on WMSDs in the automotive industry over the past 5 years (2020-2025)

Author/year	Title	Study design	Objective	Main findings
He et al., 2023 [12]	Prevalence of work-related musculoskeletal disorders among workers in the automobile manufacturing industry in China: a systematic review and meta-analysis	Systematic review and meta-analysis	To assess the prevalence of WMSDs among workers in the automobile manufacturing industry in China	The overall 12-month prevalence of WMSDs was 53.1%. The most affected region was the lower back/waist (36.5%), followed by the neck, shoulders, upper back, elbows, wrists/hands, buttocks/legs, knees, and ankles/feet
Marçal et al., 2020 [13]	Riscos ergonômicos de uma linha de produção na indústria automotiva	Cross-sectional observational study	To identify ergonomic risks in an assembly line of an automotive company specialized in welding metal components for aircraft, trucks, tractors, and automobiles	A high frequency of musculoskeletal symptoms was observed, especially in the shoulders (17% in the past 7 days), right wrist (17% in the past 12 months), wrists (20% in the past 7 days), and lower back (27% in the past 12 months and in the past 7 days)
Luo et al., 2022 [14]	Prevalence of occupational musculoskeletal disorders among workers in heavy-duty automobile parts factories in Beijing Municipality	Cross-sectional observational study with convenience sampling	To estimate the prevalence of WMSDs and analyze associated factors among workers in heavy-duty automobile parts manufacturing companies in Beijing	A total of 264 workers were evaluated. The prevalence of WMSDs was 70.08%, with higher involvement of the lower back/waist (41.28%), shoulders (40.15%), neck (39.02%), and upper back (33.33%)
Chen et al., 2021 [15]	Analysis on prevalence status and the influencing factors of work-related musculoskeletal disorders among workers in an automobile manufacturing enterprise in Guangzhou City	Descriptive and analytical observational study	To analyze the prevalence and associated factors of WMSDs among workers in an automobile manufacturing enterprise in Guangzhou	The detection rate of WMSDs was 43.9%. The most affected regions were the neck (24.5%), shoulders (21.1%), ankles (20.1%), and lower back (16.7%)
Chen et al., 2023 [16]	Analysis of work-related musculoskeletal disorders among automobile manufacturing logistics workers in Guangzhou	Cross-sectional observational study with convenience sampling	To investigate the occurrence of WMSDs among logistics workers in the automotive industry, identifying categories and influencing factors	The overall prevalence of WMSDs was 42.9%. The most affected regions were the neck (23.5%), shoulders (21.3%), and lower back (19.1%). In addition, 69.0% of workers had simultaneous involvement of two or more body segments
Li et al., 2024 [17]	Study on risk prediction model of neck work-related musculoskeletal disorders among automobile manufacturing enterprise workers	Cross-sectional observational study with cluster convenience sampling	To identify risk factors for cervical WMSDs and develop a risk prediction model	A total of 1,783 workers were interviewed, with an incidence of cervical WMSD symptoms of 24.8%. Older age, female sex, smoking, uncomfortable postures, repetitive head movements, occupational stress, and conflicting tasks increased the risk of cervical symptoms

WMSDs = work-related musculoskeletal disorders.

## DISCUSSION

The analysis of scientific production over the past 5 years reveals a consistent pattern of concern regarding WMSDs in the automotive sector, particularly in activities performed on assembly lines. The reviewed studies converge in indicating that, despite regulatory advances and the implementation of ergonomic initiatives, WMSDs continue to represent a substantial burden for workers as well as for productive and economic systems.

He et al. [^12^] examined the prevalence of WMSDs in the Chinese automotive industry through a systematic review and meta-analysis, highlighting the magnitude of these conditions within the sector. The authors report that, in 2016, occupational risks accounted for 76.1 million cases of disability worldwide, of which 20.3% were associated with occupational ergonomic factors. In addition, in 2019, WMSDs resulted in the loss of 5.5 million disability-adjusted life years, with a significant impact on the global young population. The study further emphasizes that automotive industry workers are particularly exposed to risk factors such as repetitive movements, inadequate postures, and physical overload, conditions that favor the occurrence of WMSDs. These findings reinforce the need for continuous prevention strategies and occupational health surveillance specifically targeted to this sector, given the high burden of disease associated with working conditions.

In the study by Marçal et al. [^13^], the distribution of musculoskeletal symptoms suggests a pattern of involvement closely linked to the ergonomic demands of the automotive industry. The high frequency of pain in the shoulders, wrists, and lower back indicates biomechanical overload, possibly related to the adoption of inadequate postures and repetitive movements. The persistence of symptoms both over a 7-day period and across 12 months underscores the need to implement preventive strategies and adjustments in work processes to reduce exposure to risk factors and mitigate adverse effects on workers’ health.

Complementarily, Soares et al. [^3^], in their analysis of reported cases of RSIs and WMSDs in the federal public service, also demonstrate the persistence of these conditions across different occupational contexts in Brazil. The authors highlight relevant functional and psychosocial impacts, reinforcing the importance of integrated worker health surveillance and prevention actions. The presence of these conditions in diverse work settings suggests a pattern of illness strongly associated with work organization and the ergonomic demands of performed activities.

Luo et al. [^14^] further reinforce this trend by identifying a WMSD prevalence of 70.08% among workers in heavy-duty vehicle parts factories in Beijing. The study indicates that the most affected regions were the lower back, shoulders, and upper back, evidencing the close relationship between physical workload and ergonomic aspects in the development of WMSDs. In addition, the authors emphasize that the type of activity performed, posture adopted throughout the workday, and task duration are determining factors for the occurrence of these conditions.

Based on the findings of Luo et al. [^14^], it can be observed that WMSDs in the automotive sector do not arise exclusively from adverse physical conditions, but also reflect structural weaknesses in organizational processes. The study shows that psychosocial factors, such as productivity pressure, accelerated work pace, and lack of regular breaks, may intensify the effects of biomechanical demands and contribute to illness. The combination of physical overload and organizational stressors creates a context conducive to the chronicity of musculoskeletal symptoms, with repercussions that extend beyond the physical dimension to workers’ mental and social well-being.

The high prevalence of these conditions among automotive sector workers points to the need for strategies focused on ergonomic adaptation and restructuring of working conditions, with the aim of reducing physical overload and minimizing health impacts. In this regard, it is recommended that interventions adopt an integrated approach, addressing not only the physical aspects of work but also the psychosocial factors involved in labor activities.

The study by Chen et al. [^15^], which evaluated 7,065 workers from an automobile manufacturing plant in Guangzhou across multiple occupational roles, identified a WMSD prevalence of 43.9%, reinforcing the relevance of these conditions in the automotive industry. The distribution of affected body regions suggests that the physical and postural demands inherent to the performed activities play a central role in the occurrence of WMSDs. The study highlights that factors such as inadequate postures and repetitive movements are directly associated with the development of these conditions, underscoring the importance of ergonomic assessments and the implementation of workplace interventions. In addition to the high prevalence of WMSDs, the study demonstrated that their occurrence varies according to the nature of the tasks performed, with higher concentration among workers engaged in short-cycle tasks and accelerated work pace. This finding indicates that, beyond repetitiveness and inadequate postures, the imposed production rhythm constitutes an aggravating factor in the development of WMSDs. These results reinforce that preventive strategies should consider work organization and task execution times, promoting adequate breaks and redistribution of effort to reduce ergonomic risks and support health promotion within industrial work environments and processes.

Another study conducted by Chen et al. [^16^] investigated 1,442 workers performing a specific function, logistics, in two automobile manufacturing companies located in the same city, again demonstrating a substantial prevalence of WMSDs (42.9%) among workers. Among the main associated risk factors, inadequate postures during task performance and repetitive movements were prominent. These findings reinforce the complexity of WMSD prevention in the automotive sector and point to the need for more effective and sustainable ergonomic interventions in work environments. The study also highlights the importance of detailed mapping of job functions within logistics operations, showing that different stages of the production process present varying levels of WMSD risk. This differentiation indicates that preventive actions should be specifically targeted, considering the particularities of each task and its biomechanical demands. A segmented analysis of working conditions not only enables the identification of critical exposure points but also supports the development of more focused intervention programs, ranging from organizational adjustments to workstation design modifications, thereby enhancing the effectiveness of ergonomic risk reduction.

The influence of ergonomic factors on the occurrence of WMSDs is also emphasized in the study by Li et al. [^17^], in which repetitive movements, uncomfortable postures, and prolonged maintenance of the neck in a fixed position were identified as risk predictors for the development of WMSDs. These findings supported the construction of a predictive risk model that integrates ergonomic variables and workplace-specific factors to estimate the probability of cervical disorders among automotive industry workers. This approach provides a more detailed understanding of individual and contextual factors associated with WMSDs, allowing for more accurate assessment of occupational risks. The application of predictive models of this nature may represent a relevant tool for guiding more targeted and effective ergonomic interventions, contributing to the reduction of occupational disease incidence based on job and worker characteristics.

The evidence gathered in this review reinforces that prolonged static postures, repetitive movements, and intense production pace remain central elements in the onset and persistence of these occupational conditions, which are subject to compulsory notification in Brazil. In this context, the importance of implementing established preventive strategies, such as active breaks, workplace exercise programs, ergonomic training, and workstation adjustments, is highlighted.

However, there is a clear need for interventional studies that incorporate innovative and participatory approaches, ensuring the effective inclusion of workers at all stages of the process. Such investigations should go beyond the analysis of WMSDs and their organizational determinants, also encompassing the development, evaluation, and implementation of technologies aimed at reducing physical strain in work environments and processes.

In this regard, the promotion of network-based research conducted by universities and public teaching and research institutions is emphasized, with a focus on generating applied knowledge and developing integrated solutions aligned with Visat guidelines and the concrete realities of the world of work. Furthermore, Chapter III - Strategies, item VII, of PNSTT [^9^] highlights the importance of support, across all three levels of management, for the development of studies and research that inform actions for health promotion, protection, recovery, and rehabilitation within work environments and processes.

By integrating research, development, and technological innovation with worker protagonism and Visat principles, the potential for more effective interventions is expanded, contributing to the consolidation of safer and healthier work environments. Such actions may also generate positive impacts on productivity and economic outcomes in the automotive sector by reducing work absences and improving working conditions.

## CONCLUSIONS

The findings indicate that the conduct of interventional studies that also evaluate innovative prevention and management strategies is strongly recommended. The adoption of evidence-based interventions has the potential to advance efforts to address and reduce the impact of WMSDs on workers’ health, promote improvements in work organization, and enhance occupational risk control. These initiatives contribute to the development of safer, healthier, and more sustainable work environments within the automotive industry.

## Data Availability

The data supporting the findings of this study are available within the article.
